# Integrative analysis of the choroid by quantifying Haller vessel and choriocapillaris parameters in different drusen subtypes

**DOI:** 10.1038/s41598-021-94627-1

**Published:** 2021-07-30

**Authors:** Hyungwoo Lee, Seungmin Kim, Myung Ae Kim, Young Joon Jo, Woo Hyuk Lee, Hyung Chan Kim, Hyewon Chung

**Affiliations:** 1 Department of Ophthalmology, Konkuk University School of Medicine, Konkuk University Medical Center, 120-1 Neungdong-ro, Gwangjin-gu, Seoul, 05030 Republic of Korea; 2 Department of Ophthalmology, Chungnam National University College of Medicine, Chungnam National University Hospital, Daejeon, Republic of Korea

**Keywords:** Eye diseases, Macular degeneration

## Abstract

This study aimed to quantify the Haller vessel and choriocapillaris (CC) parameters in drusen subtypes in nonexudative age-related macular degeneration (AMD) and pachydrusen. Ninety-five eyes of 80 patients and 28 control eyes were categorized into soft drusen, subretinal drusenoid deposit (SDD), soft drusen plus SDD, pachydrusen, and control groups. The diameter, length and intersections of Haller vessels and the total area, size and number of CC flow voids were quantified using en face optical coherence tomography (OCT) or OCT angiography. The pachydrusen group showed the largest Haller vessel area and diameter and shortest total length but similar CC parameters to those in the control group. The soft drusen plus SDD group showed the largest CC flow void area and size, while the Haller parameters were similar to those in the control group. The area and size of the flow voids in the SDD group were smaller than those in the soft drusen plus SDD group. Based on unsupervised machine learning, the eyes were classified into 4 clusters—the control, pachydrusen, soft drusen plus SDD and soft drusen plus SDD groups. Cluster 3 showed a larger diameter and shorter total length of the Haller vessels than cluster 4.

## Introduction

Drusen are an important phenotype of age-related macular degeneration (AMD). Recent developments in imaging techniques have further differentiated drusen into soft drusen, subretinal drusenoid deposits (SDDs), and pachydrusen^1–3^.

Although the pathogenesis of nonexudative AMD is unclear, choroidal changes are thought to be important in the development of drusen deposits. Features of gross choroidal morphology, including choroidal thickness (CT), choroid vascularity index (CVI), and flow voids in the choriocapillaris (CC), have been investigated in each type of drusen^4–6^. The CVI has an advantage in that it represents the proportion of the luminal space of the choroid^7^. SDDs demonstrate a lower mean CT/CVI than eyes with no AMD or early AMD with soft drusen^5^. Similarly, in this cohort followed for 24 months, SDD showed a lower ratio of luminal and total choroidal area compared to soft drusen and control eyes at both baseline and month 24^8^. Contradictory results have also been reported, showing that the CVI is significantly lower in AMD eyes without SDDs than in those with SDDs^9^. This discrepancy among studies might originate from the innate limitation of the CVI because choroidal vascular components, including the CC, Sattler and Haller layers, congregate in a single B-scan, generating a mixed effect of the 3 choroidal layers^7^. Among the three layers, the Haller layer is a major component of the choroidal vasculature in terms of the total area and emerges as an important phenotype in specific drusen (e.g., pachydrusen) and other diseases, including pachychoroid diseases. En face OCT has advantages in the ability to represent the detailed en face morphology of Haller vessels, enabling quantification of vessel morphology, including the vascular area and diameter, number of vascular intersections, and branch length^10,11^. In the CC layer, flow voids observed on optical coherence tomography angiography (OCTA) are increased in nonexudative AMD patients compared with healthy controls^6,12^. Additionally, the CC flow void area is associated with the progression of geographic atrophy (GA) and neovascular AMD (nAMD)^13–19^. However, little is known about the clinical implications based on the integrated information from both large choroidal vascular morphology and CC flow in nonexudative AMD.

Moreover, the specific characteristics of the mixed type of soft drusen and SDDs (soft drusen plus SDDs) and its association with the progression of GA is still ambigous^15,20^. Lee et al. reported that soft drusen plus SDDs showed the highest risk for macular neovascularization (MNV) development in the contralateral eye followed by the soft drusen only and SDD only groups, suggesting a devastating effect of nAMD development in the mixed type of drusen^15^. For GA, the specific risk of soft drusen plus SDD for the progression of GA is not known well, although both the soft drusen only and SDD only groups are associated with GA^21–23^. Therefore, the analysis of each drusen subtype, including mixed type based on both CC flow and Haller vessel morphology analyzed together, might unveil the specific characteristics of the choroidal environment in each subtype of drusen.

In the current study, we quantified various en face morphological parameters of the Haller and CC layers in the following subgroups: the control, soft drusen-only, SDD-only, soft drusen plus SDD, and pachydrusen groups. From en face OCT images of the Haller vessels, the vessel diameter (mean and standard deviation [SD], maximum), vessel length (total vessels and branch vessels), and number of vessel intersections were calculated. From CC flow voids in OCTA images, the total area, average size and number of flow voids were analyzed. The quantified parameters were compared among the 5 subgroups. Additionally, we applied an unsupervised machine learning (ML) algorithm based on these quantified parameters to obtain data-driven clusters.

## Results

### Baseline characteristics

**Figure 1:**
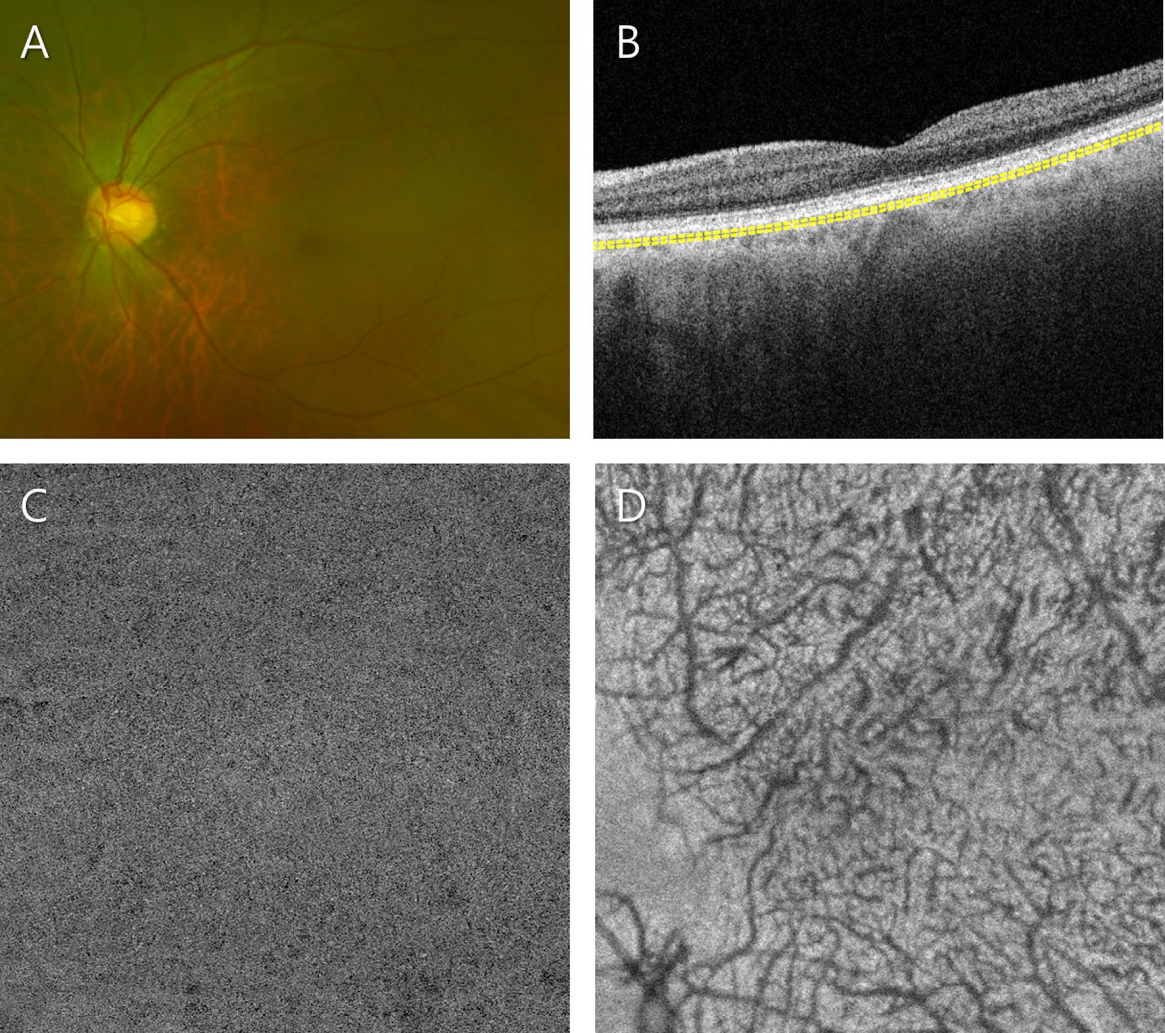
Representative subject in the healthy control group. (**A**) Ultra-widefield fundus photograph showing no specified lesions. (**B**) Optical coherence tomography (OCT) B-scan image showing no pathological changes. The subfoveal choroidal thickness was 153 µm. Slab to acquire the choriocapillaris (CC) flow is depicted as a yellow dashed line. (**C**) OCT angiography (OCTA) image of the CC. Relatively small flow voids were evenly distributed throughout the scanned area. The average size, number and total area of the flow voids were 221.1 µm^2^, 27,209, and 6.0 mm^2^, respectively. (**D**) Structural en face OCT image of the Haller layer. The mean and standard deviation of the Haller vessel diameter were 73.8 and 31.3 µm, respectively.

**Figure 2:**
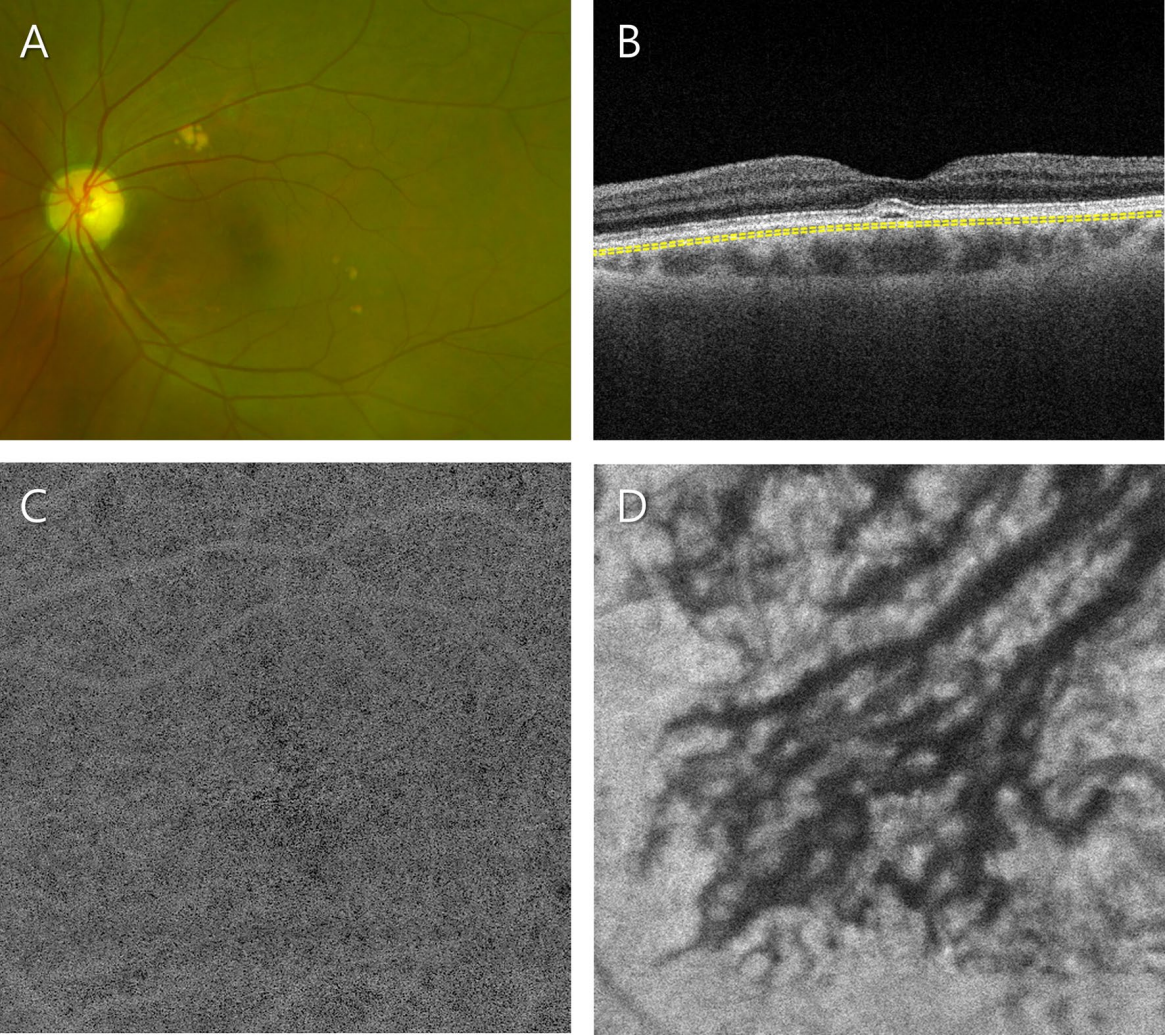
Representative patient in the pachydrusen group. (**A**) Ultra-widefield fundus photograph showing scattered yellow deposits with well-defined boundaries. (**B**) Optical coherence tomography (OCT) B-scan image showing an increased choroidal thickness with dilated Haller vessels. The subfoveal choroidal thickness was 275 µm. Slab to acquire the choriocapillaris (CC) layer is depicted as a yellow dashed line. (**C**) OCT angiography (OCTA) image of the CC. The characteristics of the flow voids were similar to those in the control group in that the relatively small flow voids were evenly distributed throughout the scanned area, while sparse focal defects were observed. The average size, number and total area of flow voids were 217.9 µm^2^, 26,831, and 6.1 mm^2^, respectively. (**D**) Structural en face OCT image of the Haller layer. Dilated Haller vessels are prominent. The mean and standard deviation of the Haller vessel diameter were 141.8 and 79.3 µm, respectively.

**Figure 3:**
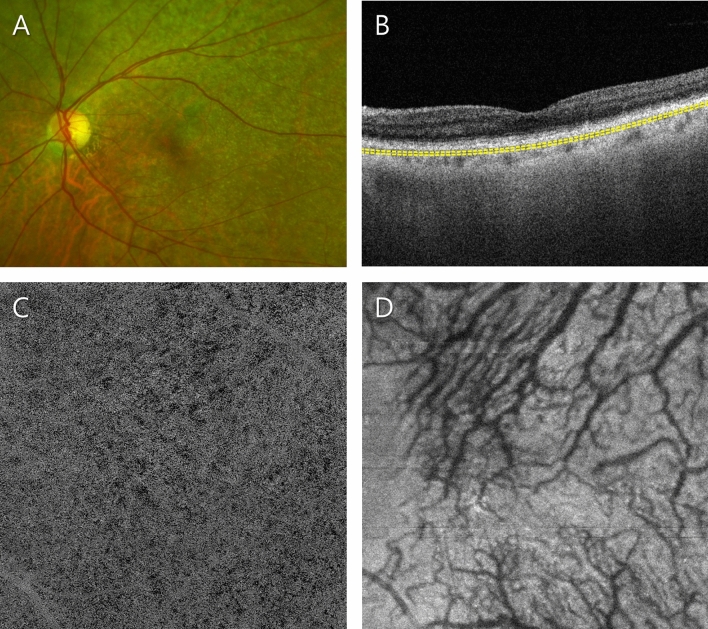
Representative patient in the soft drusen plus subretinal drusenoid deposit (SDD) group. (**A**) Ultra-widefield fundus photograph showing drusen mixed with dispersed SDD at the posterior pole. (**B**) Optical coherence tomography (OCT) B-scan image showing the mixed form of drusen and SDD. The subfoveal choroidal thickness was 153 µm. Slab to acquire the choriocapillaris (CC) layer is depicted as a yellow dashed line. (**C**) OCT angiography (OCTA) image of the CC. Enlarged flow voids were distributed heterogeneously throughout the scanned area. The average size, number and total area of flow voids were 684.6 µm^2^, 17,360, and 11.9 mm^2^, respectively. (**D**) Structural en face OCT image of the Haller layer. The mean and standard deviation of the Haller vessel diameter were 86.8 and 38.8 µm, respectively.

**Figure 4:**
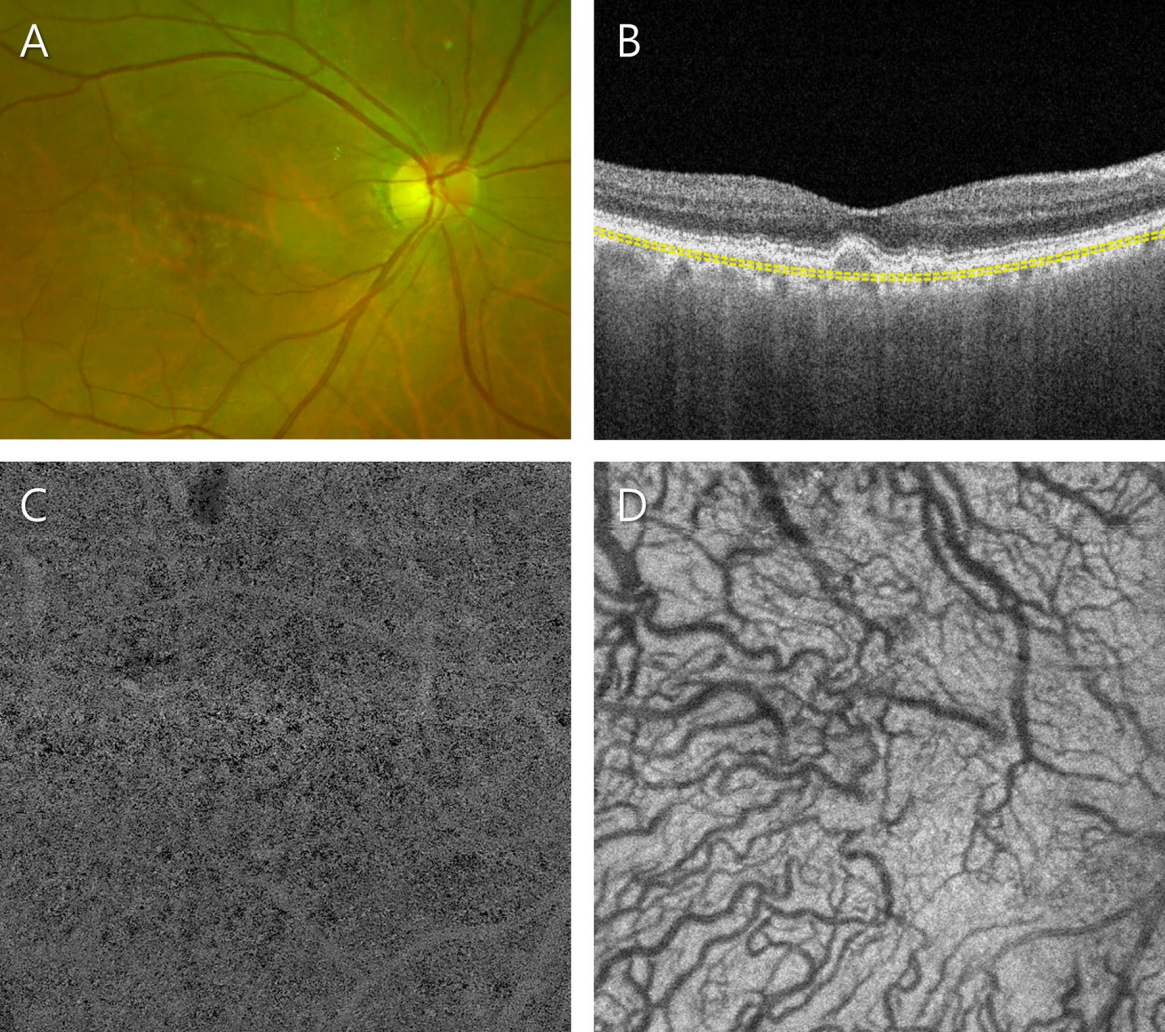
Representative case of a patient in the soft drusen-only group. (**A**) Ultra-widefield fundus photograph showing soft drusen at the macula. (**B**) Optical coherence tomography (OCT) B-scan image. The subfoveal choroidal thickness was 99 µm. Slab to acquire the choriocapillaris (CC) layer is depicted as a yellow dashed line. (**C**) OCT angiography (OCTA) image of the CC. Multiple enlarged flow voids were distributed throughout the scanned area. The average size, number and total area of the flow voids were 369.5 µm^2^, 22,904, and 8.5 mm^2^, respectively. (**D**) Structural en face OCT image of the Haller layer. The mean and standard deviation of the Haller vessel diameter were 77.1 and 33.4 µm, respectively.

**Figure 5:**
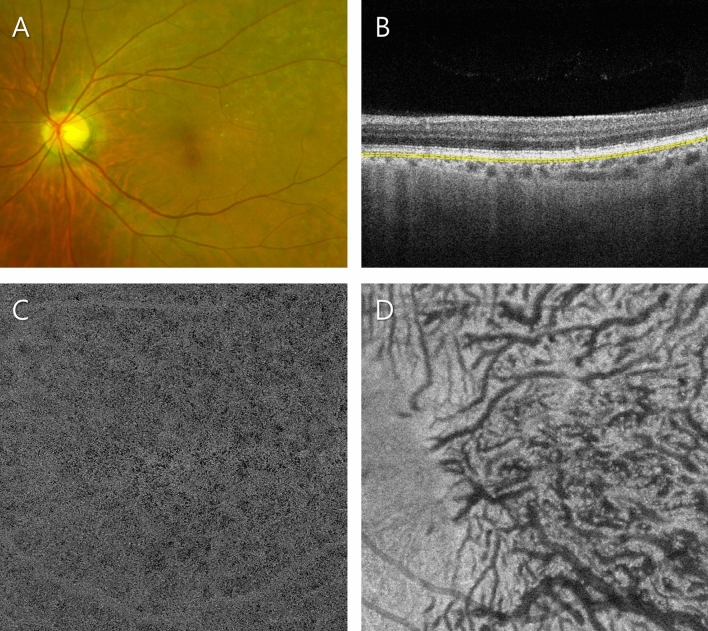
Representative patient in the subretinal drusenoid deposit (SDD)-only group. (**A**) Ultra-widefield fundus photograph showing the dispersed SDD at the superotemporal side. (**B**) Optical coherence tomography (OCT) B-scan image. The subfoveal choroidal thickness was 221 µm. Slab to acquire the choriocapillaris (CC) layer is depicted as a yellow dashed line. (**C**) OCT angiography (OCTA) image of the CC. Enlarged flow voids were notable in all subgroups. The average size, number and total area of flow voids were 317.9 µm^2^, 24,256, and 7.7 mm^2^, respectively. (**D**) Structural en face OCT image of the Haller layer. The mean and standard deviation of the Haller vessel diameter were 84.7 and 36.7 µm, respectively.

In total, 129 eyes of 105 consecutive subjects were enrolled. Among them, 6 patients were excluded because of poor-quality OCTA images, and 123 eyes of the other 103 subjects were included in this study. The numbers of eyes in the control, soft drusen-only, SDD-only, soft drusen plus SDD and pachydrusen groups were 28 (22.8%), 18 (15.1%), 21 (17.6%), 37 (31.1%), and 19 (16.0%), respectively. The baseline characteristics are presented in Table 1, and representative images of the 5 subgroups are represented in Figs. 1, 2, 3, 4 and 5.

**Table 1:** Clinical characteristics and quantitative parameters of patients according to drusen type.

	Control (N = 28)	Pachydrusen (N = 19)	SDD only (N = 21)	Soft drusen only (N = 18)	Soft drusen plus SDD (N = 37)	P*
Patient number	23	16	18	17	29	
Age (year), mean ± SD	67.2 ± 10.6	65.1 ± 7.2	72.5 ± 7.5	75.1 ± 5.7	72.9 ± 6.7	< 0.001^†^
Male/female (%)	10/18	11/8	6/15	14/4	8/29	0.001
Visual acuity (LogMAR)	0.10 ± 0.18	0.06 ± 0.12	0.08 ± 0.12	0.07 ± 0.08	0.08 ± 0.11	0.85
**Haller’s vessels**						
Diameter, mean (μm)	83.9 ± 8.0	113.4 ± 15.9	85.8 ± 12.9	84.7 ± 12.2	87.1 ± 10.9	< 0.001^†^
Diameter, SD (μm)	36.8 ± 5.8	59.9 ± 12.1	38.1 ± 9.9	38.4 ± 8.9	39.3 ± 13.6	< 0.001^†^
Diameter, maximum (μm)	290.6 ± 46.8	400.4 ± 48	299.4 ± 61.9	325.3 ± 82.8	326.9 ± 71.9	< 0.001^†^
Total vessel length (mm)	58.2 ± 7.2	43.3 ± 6.9	53.5 ± 8.0	53.8 ± 9.6	51.3 ± 11.8	< 0.001^†^
Total vessel area (mm^2^)	8.8 ± 1.4	10.5 ± 2.2	8.1 ± 1.8	8.5 ± 1.2	8.2 ± 1.5	< 0.001^†^
Number of intersections	147.8 ± 34.6	121.4 ± 49.1	117.7 ± 34.1	132.2 ± 37.3	132.5 ± 51.5	0.02^†^
Branch vessel length, mean (μm)	210.9 ± 33.7	244.8 ± 26.9	231.6 ± 29.1	222 ± 37.3	227 ± 32.1	0.01^†^
Branch vessel length, SD (μm)	222.9 ± 54.2	244.7 ± 45.5	260 ± 43.0	235.7 ± 57.0	247.7 ± 56.0	0.11
**Choriocapillaris flow voids**						
Average size (μm^2^)	228.1 ± 61.1	251.6 ± 34.3	321.1 ± 132.4	342.8 ± 102.5	406.7 ± 135.9	< 0.001^†^
Number	25,263.4 ± 2102.3	25,911.3 ± 445.8	24,201.3 ± 2,530.5	23,511.3 ± 2,873.3	22,522.5 ± 2,778.9	< 0.001^†^
Total flow void area (mm^2^)	5.8 ± 1.6	6.5 ± 0.8	7.5 ± 1.7	7.9 ± 1.7	8.8 ± 1.7	< 0.001^†^
Subfoveal CT (µm)	210.9 ± 75.6	318.3 ± 89.1	146.8 ± 61.0	158.7 ± 69.6	148.1 ± 50.7	< 0.001^†^

All values are presented as the mean ± standard deviation (SD).

*SDD* subretinal drusenoid deposit *LogMAR*, Logarithm of the minimum angle of resolution *CT*, choroidal thickness.

*P-value from Kruskal–Wallis test for all 5 subgroups (sex ratio was compared by chi-square test).

^†^Significant difference by Kruskal–Wallis test.

### Differences in characteristics among diseases grouped by conventional diagnosis

The Haller vessel parameters were compared among the 5 disease subgroups. The pachydrusen group showed a larger CT, larger area and larger Haller vessel diameter, including the mean, SD, and maximum, than the other 3 drusen groups (Table 1, Supplementary Table S1). Compared with the control group, only the pachydrusen group showed a significant increase in the Haller vessel diameter (mean, SD, maximum) (Supplementary Table S1, Figs. 1, 2). These differences were also observed between the pachydrusen and other drusen subgroups (Supplementary Table S1, Figs. 3, 4, 5). The pachydrusen group showed the shortest total Haller vessel length (Supplementary Table S1). No significant difference was observed in Haller vessel parameters among the 3 other drusen groups, including the soft drusen, SDD and soft drusen plus SDD groups (Supplementary Table S1, Figs. 3, 4, 5).

When the CC parameters were compared among the 5 subgroups, the pachydrusen group showed a smaller total area and average size of flow voids than the drusen and soft drusen plus SDD groups but showed no significant difference compared with the SDD group (Supplementary Table S1). Compared with the control group, the pachydrusen group showed no difference in CC parameters, while the other 3 drusen groups showed a larger total area and average size of flow voids (Supplementary Table S1).

The soft drusen plus SDD group showed a larger total flow void area and average flow void size than the SDD group (Supplementary Table S1, Figs. 3, 5).

### ML-mediated clustering based on the quantified parameters

Silhouette analysis suggested that dividing patients into 4 groups was most appropriate (Supplementary Fig. S2). By applying k-means clustering, the patients were divided into 4 clusters: cluster 1 (49 eyes of 41 subjects), cluster 2 (37 eyes of 30 subjects), cluster 3 (13 eyes of 11 subjects) and cluster 4 (24 eyes of 21 subjects) (Table 2).

**Table 2:** Distribution of each drusen type in 4 clusters.

	Cluster 1 (N = 49)	Cluster 2 (N = 37)	Cluster 3 (N = 13)	Cluster 4 (N = 24)	P
Healthy control (%)	20 (40.8)	6 (16.2)	0 (0)	2 (8.3)	< 0.001*
Soft drusen only (%)	6 (12.2)	4 (10.8)	3 (23.1)	5 (20.8)	0.54
SDD only (%)	10 (20.4)	5 (13.5)	2 (15.4)	4 (16.7)	0.86
Soft drusen plus SDD (%)	12 (24.5)	4 (10.8)	8 (61.5)	13 (54.2)	< 0.001*
Pachydrusen (%)	1 (2)	18 (48.6)	0 (0)	0 (0)	< 0.001*

*SDD* subretinal drusenoid deposit.

*Significant difference among the 4 groups using the chi-square test.

### Differences in characteristics among the 4 clusters

The disease groups showing the highest frequency in clusters 1 and 2 were the control and pachydrusen, respectively. In both clusters 3 and 4, the soft drusen plus SDD group was the major component (Table 2). The drusen-only and SDD-only groups were distributed among all 4 clusters (Table 2).

Differences in characteristics among the 4 clusters are summarized in Table 3 and Supplementary Table S2. Cluster 2, which was dominated by pachydrusen, showed a younger age, the largest Haller vessel diameter (mean, maximum and SD), and the largest total vessel area. The diameters of the Haller vessels in cluster 3 were smaller than those in cluster 2 but larger than those in cluster 4 (Table 3, Supplementary Table S2). Clusters 2 and 3 showed a shorter total length and longer branch length of the Haller vessels than clusters 1 and 4 (Table 3, Supplementary Table S2).

**Table 3:** Summary of differences in characteristics among the 4 clusters.

	Cluster 1 (N = 49)	Cluster 2 (N = 37)	Cluster 3 (N = 13)	Cluster 4 (N = 24)	P*	P 1 vs 2	P 1 vs 3	P 1 vs 4	P 2 vs 3	P 2 vs 4	P 3 vs 4
Age (year), mean ± SD	70.8 ± 8.7	66.3 ± 7.7	75.2 ± 7.1	74.6 ± 6.8	< 0.001*	0.006^†^	0.19	0.11	0.001^†^	< 0.001^†^	0.84
Male/female	20/29	16/21	2/11	11/13	0.28	0.82	0.07	0.84	0.09	0.68	0.06
Visual acuity (LogMAR)	0.06 ± 0.13	0.09 ± 0.15	0.11 ± 0.10	0.09 ± 0.11	0.07	0.08	0.01	0.08	0.17	0.71	0.46
**Haller’s vessels**											
Diameter, mean (μm)	81.4 ± 6.5	106.9 ± 15.2	90.4 ± 11.9	80.4 ± 5.3	< 0.001*	< 0.001^†^	0.003^†^	0.48	< 0.001^†^	< 0.001^†^	0.002^†^
Diameter, SD (μm)	34.7 ± 3.6	54.9 ± 12.8	43.6 ± 18.6	34.0 ± 2.9	< 0.001*	< 0.001^†^	0.007^†^	0.54	< 0.001^†^	< 0.001^†^	0.002^†^
Diameter, maximum (μm)	281.6 ± 44.3	388.5 ± 56.5	348.9 ± 97.8	303.0 ± 46.8	< 0.001*	< 0.001^†^	0.007^†^	0.05	0.03	< 0.001^†^	0.14
Total vessel length (mm)	56.8 ± 7.0	45.6 ± 8.4	42.7 ± 12.0	59.0 ± 7.5	< 0.001*	< 0.001^†^	< 0.001^†^	0.37	0.65	< 0.001^†^	< 0.001^†^
Total vessel area (mm^2^)	8.2 ± 1.3	9.9 ± 2.0	7.1 ± 1.6	8.8 ± 1.0	< 0.001*	< 0.001^†^	0.04	0.06	< 0.001^†^	0.03	0.001^†^
Number of intersections	140.3 ± 34.3	115.1 ± 40	83.7 ± 22.5	165.6 ± 41.7	< 0.001*	0.001^†^	< 0.001^†^	0.03	0.002^†^	< 0.001^†^	< 0.001^†^
Branch vessel length, mean (μm)	208.4 ± 24.8	251.1 ± 24.4	265.4 ± 16.6	202.6 ± 20.3	< 0.001*	< 0.001^†^	< 0.001^†^	0.31	0.07	< 0.001^†^	< 0.001^†^
Branch vessel length, SD (μm)	222.7 ± 39.9	263.8 ± 48.2	315.7 ± 29.1	207.7 ± 39.3	< 0.001*	< 0.001^†^	< 0.001^†^	0.11	0.001^†^	< 0.001^†^	< 0.001^†^
**Choriocapillaris flow voids**											
Average size (μm^2^)	252.8 ± 56.8	256.8 ± 45.4	491.9 ± 148.3	452.0 ± 107.3	< 0.001*	0.79	< 0.001^†^	< 0.001^†^	< 0.001^†^	< 0.001^†^	0.50
Number	25,174.3 ± 1,580.9	25,732.7 ± 951.4	20,214.1 ± 2,820.0	21,500.8 ± 2,151.5	< 0.001*	0.19	< 0.001^†^	< 0.001^†^	< 0.001^†^	< 0.001^†^	0.19
Total flow void area (mm^2^)	6.4 ± 1.4	6.6 ± 1.0	9.7 ± 1.9	9.5 ± 1.0	< 0.001*	0.80	< 0.001^†^	< 0.001^†^	< 0.001^†^	< 0.001^†^	0.52
Subfoveal CT (µm)	165.5 ± 73.2	271.4 ± 87.2	141.6 ± 63.4	140.7 ± 46.9	< 0.001*	< 0.001^†^	0.38	0.25	< 0.001^†^	< 0.001^†^	0.94

*SD* standard deviation, *CT* choroidal thickness.

*Significant difference by Kruskal–Wallis test (sex ratio was compared by chi-square test).

^†^Significant difference by Mann–Whitney test with Bonferroni’s correction (P < 0.008).

The total area and average size of the flow voids in the CC layer were larger in clusters 3 and 4 than in clusters 1 and 2, while the number of flow voids was smallest in clusters 3 and 4. No significant difference was found in these CC parameters between clusters 1 and 2 and between clusters 3 and 4 (Table 3, Supplementary Table S2).

When only the soft drusen plus SDD eyes in clusters 3 and 4 were compared, cluster 3 showed a larger diameter, larger area and shorter total length of the Haller vessels than cluster 4, while the CC-associated parameters were not different (Supplementary Table S3).

## Discussion

In the present study, we conducted an integrative analysis of the choroid to discover different characteristics of drusen subtypes, including a mixed subtype. To more precisely reveal properties of the choroid, we simultaneously quantified the various morphologic parameters of en face Haller vessel and CC flow voids. As a result, the pachydrusen and soft drusen plus SDD groups were distinguished from the other drusen subgroups. Additionally, we applied unsupervised ML based on the quantified parameters, resulting in 4 clusters. Clusters 1 and 2 primarily comprised healthy control and pachydrusen eyes, respectively. Both clusters 3 and 4 primarily comprised soft drusen plus SDD eyes, and clusters 3 and 4 were distinguished from each other by the diameter and length of the Haller vessels.

The pachydrusen group, which dominated cluster 2, showed larger values for the area and diameter (mean, SD and maximum) of the Haller vessels than the other subgroups. Regarding the CC parameters, the pachydrusen group showed no significant differences compared with the healthy control group. The pachydrusen group was associated with thick choroid with pachyvessels and choroidal hyperpermeability^3^. In a study of the incidence of nAMD in the fellow eye according to the drusen type^15^, no significant difference was found between the pachydrusen and no drusen groups. Our results also suggest that the flow of the CC in the pachydrusen group was similarly well preserved as that in the control group, leading to a nonsignificant increase in the risk of advanced AMD. The preserved CC, despite the presence of ‘’pachyvessels’’ in the pachydrusen group and cluster 2, might be attributed to the distinct characteristics of Haller vessels with the shortest total length and largest diameter. According to Poiseuille’s law, vessel resistance is directly proportional to the length and inversely proportional to the radius to the fourth power^24^. Therefore, Haller vessels with the shortest total length and largest diameter might increase blood flow and facilitate perfusion to the CC layer. Although the definition of pachychoroid spectrum diseases remains debatable, these results suggest that a thick choroid and/or pachyvessels are not necessarily related to pathological conditions for at least a certain period before any other factors become involved.

The soft drusen plus SDD group showed a larger total area and average size of flow voids in the CC than the pachydrusen and healthy control groups. Clusters 3 and 4, of which the drusen plus SDD group was dominant, showed larger flow voids in the CC than clusters 1 and 2. Additionally, the soft drusen plus SDD group showed larger flow voids in the CC than the SDD group. Compared with the soft drusen-only group, the soft drusen plus SDD group also showed a larger flow void area, although the difference was not significant. We speculate that the largest flow void in the CC of the soft drusen plus SDD group is due to the additive effect of soft drusen and SDDs on CC flow voids based on the increased flow voids in these groups compared with the control group. Additionally, unsupervised clustering showed that the drusen-only and SDD-only groups were present in all three clusters in similar proportions, suggesting that the CC flow voids in the drusen-only and SDD-only groups might not be sufficient to distinguish them from the other clusters. Based on these results, the soft drusen plus SDD group might represent the most severely hypoperfused CC among all the subgroups. In a previous study^15^, the soft drusen plus SDD group showed the highest risk of MNV in the fellow eye followed by the soft drusen only and SDD only groups. However, the underlying mechanism was unclear. Additionally, women exposed to hypertensive disorders during pregnancy have an increased risk of nAMD, suggesting that ischemic events in the CC are related to nAMD development^25^. Collectively, our data show that the soft drusen plus SDD group had the largest total flow void area in the CC, likely revealing the connection with the high risk of MNV formation. On the other hand, strong evidence has not been reported regarding the specific risk of soft drusen plus SDD for the progression of GA, although both the soft drusen-only and SDD-only groups are associated with GA^21–23^. For the development and progression of GA, RPE-associated pathological conditions might be needed in addition to a CC flow deficit.

Clusters 3 and 4 were dominated by the soft drusen plus SDD group with similar CC characteristics and choroidal thicknesses, but interestingly, their Haller vessel characteristics based on the en face images were markedly different. Cluster 3 showed a similar pattern of Haller vessels as that of the pachydrusen group, namely, a larger diameter and a shorter total length, but cluster 4 showed the opposite features. This result was also repeated in the Haller vessel characteristics of the soft drusen plus SDD group in clusters 3 and 4 compared with the other clusters. Thus, drusen—particularly soft drusen plus SDD—seems heterogeneous based on the morphology of the Haller vessels. As discussed for pachydrusen, the large diameter and short total length of the Haller vessels might be beneficial for improved perfusion. Clusters 3 showed deficits in the CC flow voids similar to cluster 4. We hypothesize that the dilated Haller vessels in the limited space of the choroid in combination with a thin choroid (141.6 µm and 140.7 µm in clusters 3 and 4, respectively) might press on the CC layer above, inducing hypoperfusion as shown by the total flow void area and increased size of the flow void in the cluster 3. Alternatively, these dilated Haller vessels could be a secondary consequence of CC flow compromise. Therefore, further longitudinal studies to compare the prognosis of each type of soft drusen plus SDD according to the morphology of their Haller vessels might be needed.

The current study has the limitations of a retrospective design with a limited number of cases. In addition, in most of the eyes (77.3%), widefield fundus photography instead of conventional color photography was used. However, combined examinations with OCT was adopted for precise classification. We used built-in projection artifact removal generated from superficial retinal vessels, but this method could not completely remove the possible artifact and might hinder the accurate measurement of flow signals. Further technical improvements in removing superficial vessel masks and therefore the better analysis on the remaining area of the image are needed.

In conclusion, among 4 drusen subtypes, the distinguishing features of the pachydrusen group with a thick diameter and a short total length of Haller vessels were observed compared to controls. The soft drusen plus SDD group was differentiated by a large total area and average size of the CC flow voids. Unsupervised ML based on the above parameters identified that nonexudative AMD comprises 4 clusters: the control (1), pachydrusen (2), soft drusen plus SDDs (3 and 4). Clusters 1 and 2 were characterized by better CC perfusion compared to clusters 3 and 4. These clusters were further classified according to the features of Haller vessels. Specifically, Haller vessels exhibited larger diameters and shorter lengths in clusters 2 and 3 but not in clusters 1 and 4. A quantitative approach toward the morphology of Haller vessels and CC flow voids based on en face images might be useful to unveil the heterogeneous nature of drusen.

## Methods

### Participants

This retrospective study was approved by the Institutional Review Board (IRB) at Konkuk University Medical Center, and the requirement to obtain informed consent from the subjects was waived by the IRB (2020-02-005). All the study protocols adhered to the tenets of the Declaration of Helsinki.

We retrospectively reviewed patients with pachydrusen and nonexudative AMD, including drusen and SDDs, in the Department of Ophthalmology at Konkuk University Medical Center and Chungnam University Hospital between September 2017 and May 2020. For the controls, patients with no definite retinal disorders were selected. Eyes were excluded if they had greater than 6 diopters of myopia, a history of MNV, uveitis, glaucoma, or any other diagnosed retinal disease.

All the included patients had undergone comprehensive ophthalmological examinations, including best-corrected Snellen visual acuity, slit-lamp biomicroscopy, fundus photography, OCT (Spectralis HRA + OCT, Heidelberg Engineering, Heidelberg, Germany, at Konkuk University Medical Center and ZEISS PLEX Elite 9000, Carl Zeiss Meditech, Dublin, CA, at Chungnam National University Hospital), and OCTA (ZEISS PLEX Elite 9000, Carl Zeiss Meditech, Dublin, CA, at both institutes). The OCT volume scan comprised horizontal raster scans covering an area of 9 × 6 mm, and the OCTA scan covered an area of 6 × 6 mm. OCTA images with poor quality due to either significant motion artifacts or incorrect segmentation were further excluded.

The type of drusen was determined using color fundus photography (TRC-50DX, Topcon Corporation, Tokyo, Japan) or ultra-widefield retinal photography (Optos 200Tx; Optos plc, Dunfermline, UK) when conventional color fundus photography was unavailable, and OCT was performed according to the criteria presented in a previous study^3^. The presence of soft drusen, SDDs and pachydrusen was determined according to the criteria of previous studies^3,26^. If soft drusen was combined with at least 1 site of SDDs, the eye was categorized as having soft drusen plus SDDs. Finally, all the eyes were categorized into 5 subgroups: soft drusen only, SDD only, soft drusen plus SDD, pachydrusen, and control.

### Acquisition and interpretation of OCTA images of the CC layer

OCTA scans (6 × 6 mm) depicting CC flow were acquired using a fully automated built-in segmentation algorithm (thickness: 20 μm). This segmentation algorithm was then applied to the OCTA flow intensity data to obtain vascular images. Maximum projection analyses of the flow intensity were performed to generate en face images of the CC. Then, built-in algorithm for superficial artifact removal was applied. The total flow void area, average size and number of flow voids were analyzed using the ‘Analyze particle’ method with ImageJ software^27^. To compensate for CC signal attenuation resulting from structural changes in the RPE/Bruch membrane (BM) complex, a method described in a previous study was performed (Supplementary Fig. S1A–E)^28,29^.

### Acquisition and interpretation of the quantitative evaluation of Haller vessels

En face images of the Haller layer were obtained by locating the predefined slab of CC (20 µm in thickness) such that it spanned the center of the Haller layer in B-scans as much as possible, as described in a previous study (Supplementary Fig. S1F–H)^10^. The vessel diameter (mean, SD, and maximum), area and density, total vessel length, branch vessel length and number of intersections were quantified using the DiameterJ plugin in ImageJ as described in a previous study^30^. The subfoveal CT was measured by enhanced-depth imaging (EDI)-OCT using the built-in measuring tool^31^. Because the centerline of the Haller layer could vary according to the grader’s judgement (Supplementary Fig. S1F), the quantified morphologic parameters might also vary. Therefore, intergrader agreement was verified. Two retinal specialists (H.L. and H.C.) independently acquired the en face images of Haller vessels in randomly selected 10 eyes and calculated parameters from en face Haller vessel images. We confirmed that the intraclass correlation coefficient (ICC) were sufficiently high (Supplementary Table S4). Then, a retinal specialist (H.L.) acquired all remaining images of en face Haller vessels, and these data were confirmed by the senior retinal specialist (H.C.). If there was disagreement, a proper image of the en face Haller vessel was determined by open discussion.

### Automatic categorization of patients with MNV using ML

Cluster analysis was performed using k-means clustering, which is an unsupervised ML algorithm^32^. Before applying the k-means method, principal component analysis (PCA) was performed to reduce the dimensions of the given correlated variables^33^. The optimal number of clusters for k-means clustering was determined by silhouette analysis^34,35^.

### Statistical analysis

Differences in the variables among the groups were analyzed using the Kruskal–Wallis test. A P-value < 0.05 was considered statistically significant. Post hoc analysis was performed using Bonferroni’s correction. Comparisons of the ratio were achieved by chi-squared test. Intergrader agreements about the parameters from en face Haller vessels were evaluated by calculating ICCs. Statistical analyses were conducted using R software version 3.6. For the silhouette analysis, the “silhouette function” of the CRAN package “cluster” was used.

## Supplementary Information

Supplementary Information.

## Data Availability

The datasets generated during and/or analyzed during the current study are not publicly available due to our hospital’s policy regarding patient records but are available from the corresponding author upon reasonable request.
