# Supplementary material for: Integrative analysis of the choroid by quantifying Haller vessel and choriocapillaris parameters in different drusen subtypes
Source: Sci Rep. 2021 Jul 30;11:15509. doi: 10.1038/s41598-021-94627-1 (PMC8324802; doi:10.1038/s41598-021-94627-1)
Supplement: Supplementary file 1 — Supplementary Information. [file 41598_2021_94627_MOESM1_ESM.pdf]

Integrative analysis of the choroid by quantifying Haller vessel and choriocapillaris parameters in different drusen subtypes

Hyungwoo Lee, MD, PhD[1]; Seungmin Kim, MD[1]; Myung Ae Kim, MD[1]; Young Joon Jo, MD, PhD[2]; Woo Hyuk Lee, MD[2]; Hyung Chan Kim, MD, PhD[1]; Hyewon Chung, MD, PhD[1*]

[1]Department of Ophthalmology, Konkuk University School of Medicine, Konkuk University Medical Center, Seoul, Republic of Korea

[2]Department of Ophthalmology, Chungnam National University College of Medicine, Chungnam National University Hospital, Daejeon, Republic of Korea

Supplementary Table S1. Clinical characteristics and quantitative parameters of patients according to drusen type and their pairwise comparisons.

	Control (N=28)	Pachydrusen (N=19)	SDD only (N=21)	Soft drusen only (N=18)	Soft drusen plus SDD (N=37)	P*	P C vs P	P C vs S	P C vs D	P C vs D+S	P P vs S	P P vs D	P P vs D+S	P S vs D	P S vs D+S	P D vs D+S
Age (year), mean ± SD	67.2 ± 10.6	65.1 ± 7.2	72.5 ± 7.5	75.1 ± 5.7	72.9 ± 6.7	<0.001†	0.26	0.09	0.01	0.03	0.01	<0.001‡	<0.001‡	0.25	0.88	0.22
Male/female (%)	10/18	11/8	6/15	14/4	8/29	0.001	0.13	0.60	0.005	0.21	0.06	0.20	0.007	0.002	0.55	<0.001
Visual acuity (LogMAR)	0.10 ± 0.18	0.06 ± 0.12	0.08 ± 0.12	0.07 ± 0.08	0.08 ± 0.11	0.85	0.29	0.56	0.78	0.42	0.67	0.41	0.67	0.83	0.92	0.69
Haller's vessels																
Diameter, mean (μm)	83.9 ± 8.0	113.4 ± 15.9	85.8 ± 12.9	84.7 ± 12.2	87.1 ± 10.9	<0.001†	<0.001‡	0.89	0.77	0.36	<0.001‡	<0.001‡	<0.001‡	0.96	0.19	0.22
Diameter, SD (μm)	36.8 ± 5.8	59.9 ± 12.1	38.1 ± 9.9	38.4 ± 8.9	39.3 ± 13.6	<0.001†	<0.001‡	0.61	0.88	0.84	<0.001‡	<0.001‡	<0.001‡	0.19	0.23	0.89
Diameter, maximum (μm)	290.6 ± 46.8	400.4 ± 48	299.4 ± 61.9	325.3 ± 82.8	326.9 ± 71.9	<0.001†	<0.001‡	0.58	0.07	0.02	<0.001‡	<0.001‡	<0.001‡	0.38	0.14	0.56
Total vessel length (mm)	58.2 ± 7.2	43.3 ± 6.9	53.5 ± 8.0	53.8 ± 9.6	51.3 ± 11.8	<0.001†	<0.001‡	0.03	0.12	0.005‡	<0.001‡	0.001‡	0.002‡	0.48	0.79	0.32
Total vessel area (mm^2)	8.8 ± 1.4	10.5 ± 2.2	8.1 ± 1.8	8.5 ± 1.2	8.2 ± 1.5	<0.001†	0.01	0.06	0.39	0.20	<0.001‡	0.002‡	<0.001‡	0.21	0.30	0.73
Number of intersections	147.8 ± 34.6	121.4 ± 49.1	117.7 ± 34.1	132.2 ± 37.3	132.5 ± 51.5	0.02†	0.01	0.003‡	0.13	0.08	0.98	0.22	0.28	0.16	0.18	0.88
Branch vessel length, mean (μm)	210.9 ± 33.7	244.8 ± 26.9	231.6 ± 29.1	222 ± 37.3	227 ± 32.1	0.01†	0.001‡	0.02	0.39	0.05	0.17	0.08	0.03	0.40	0.48	0.62
Branch vessel length, SD (μm)	222.9 ± 54.2	244.7 ± 45.5	260 ± 43.0	235.7 ± 57.0	247.7 ± 56.0	0.11	0.13	0.01	0.47	0.07	0.32	0.63	0.97	0.21	0.28	0.42
Choriocapillaris flow voids																
Average size ($μm^2$)	228.1 ± 61.1	251.6 ± 34.3	321.1 ± 132.4	342.8 ± 102.5	406.7 ± 135.9	<0.001†	0.03	0.001‡	<0.001‡	<0.001‡	0.03	0.001‡	<0.001‡	0.30	0.004‡	0.07
Number	25263.4 ± 2102.3	25911.3 ± 445.8	24201.3 ± 2530.5	23511.3 ± 2873.3	22522.5 ± 2778.9	<0.001†	0.63	0.05	0.01	<0.001‡	<0.001‡	0.001‡	<0.001‡	0.57	0.01	0.17
Total flow void area (mm^2)	5.8 ± 1.6	6.5 ± 0.8	7.5 ± 1.7	7.9 ± 1.7	8.8 ± 1.7	<0.001†	0.08	0.002‡	<0.001‡	<0.001‡	0.07	0.004‡	<0.001‡	0.38	0.005‡	0.06
Subfoveal CT (μm)	210.9 ± 75.6	318.3 ± 89.1	146.8 ± 61.0	158.7 ± 69.6	148.1 ± 50.7	<0.001†	<0.001‡	0.004‡	0.046	0.001‡	<0.001‡	<0.001‡	<0.001‡	0.65	0.78	0.82

All values are presented as the mean ± standard deviation (SD); C, control; P, pachydrusen; S, subretinal drusenoid deposit; D, soft drusen; D+S, soft drusen plus SDD.
SDD, subretinal drusenoid deposit; LogMAR, logarithm of the minimum angle of resolution; CT, choroidal thickness.

* P-value from Kruskal-Wallis test for all 5 subgroups (sex ratio was compared by chi-square test).

† Significant difference by Kruskal-Wallis test.

‡ Significant difference by Mann-Whitney test with Bonferroni's correction among all 5 subgroups (P<0.005).

Supplementary Table S2. Differences in characteristics among the 4 clusters and their pairwise comparisons.

	Cluster 1 (N=49)	Cluster 2 (N=37)	Cluster 3 (N=13)	Cluster 4 (N=24)	P	P *1* vs *2*	P *1* vs *3*	P *1* vs *4*	P *2* vs *3*	P *2* vs *4*	P *3* vs *4*
Age (year), mean ± SD	70.8 ± 8.7	66.3 ± 7.7	75.2 ± 7.1	74.6 ± 6.8	<0.001*	0.006†	0.19	0.11	0.001†	<0.001†	0.84
Male/female	20/29	16/21	2/11	11/13	0.28	0.82	0.07	0.84	0.09	0.68	0.06
Visual acuity (LogMAR)	0.06 ± 0.13	0.09 ± 0.15	0.11 ± 0.10	0.09 ± 0.11	0.07	0.08	0.01	0.08	0.17	0.71	0.46
Haller's vessels											
Diameter, mean (μm)	81.4 ± 6.5	106.9 ± 15.2	90.4 ± 11.9	80.4 ± 5.3	<0.001*	<0.001†	0.003†	0.48	<0.001†	<0.001†	0.002†
Diameter, SD (μm)	34.7 ± 3.6	54.9 ± 12.8	43.6 ± 18.6	34.0 ± 2.9	<0.001*	<0.001†	0.007†	0.54	<0.001†	<0.001†	0.002†
Diameter, maximum (μm)	281.6 ± 44.3	388.5 ± 56.5	348.9 ± 97.8	303 ± 46.8	<0.001*	<0.001†	0.007†	0.05	0.03	<0.001†	0.14
Total vessel length (mm)	56.8 ± 7.0	45.6 ± 8.4	42.7 ± 12.0	59 ± 7.5	<0.001*	<0.001†	<0.001†	0.37	0.65	<0.001†	<0.001†
Total vessel area (mm^2)	8.2 ± 1.3	9.9 ± 2.0	7.1 ± 1.6	8.8 ± 1.0	<0.001*	<0.001†	0.04	0.06	<0.001†	0.03	0.001†
Number of intersections	140.3 ± 34.3	115.1 ± 40	83.7 ± 22.5	165.6 ± 41.7	<0.001*	0.001†	<0.001†	0.03	0.002†	<0.001†	<0.001†
Branch vessel length, mean (μm)	208.4 ± 24.8	251.1 ± 24.4	265.4 ± 16.6	202.6 ± 20.3	<0.001*	<0.001†	<0.001†	0.31	0.07	<0.001†	<0.001†
Branch vessel length, SD (μm)	222.7 ± 39.9	263.8 ± 48.2	315.7 ± 29.1	207.7 ± 39.3	<0.001*	<0.001†	<0.001†	0.11	0.001†	<0.001†	<0.001†
Choriocapillaris flow voids											
Average size ($μm^2$)	252.8 ± 56.8	256.8 ± 45.4	491.9 ± 148.3	452.0 ± 107.3	<0.001*	0.79	<0.001†	<0.001†	<0.001†	<0.001†	0.50
Number	25174.3 ± 1580.9	25732.7 ± 951.4	20214.1 ± 2820.0	21500.8 ± 2151.5	<0.001*	0.19	<0.001†	<0.001†	<0.001†	<0.001†	0.19
Total flow void area (mm^2)	6.4 ± 1.4	6.6 ± 1.0	9.7 ± 1.9	9.5 ± 1.0	<0.001*	0.80	<0.001†	<0.001†	<0.001†	<0.001†	0.52
Subfoveal CT (μm)	165.5 ± 73.2	271.4 ± 87.2	141.6 ± 63.4	140.7 ± 46.9	<0.001*	<0.001†	0.38	0.25	<0.001†	<0.001†	0.94

SD, standard deviation; CT, choroidal thickness.

* Significant difference by Kruskal-Wallis test (sex ratio was compared by chi-square test).

† Significant difference by Mann-Whitney test with Bonferroni's correction (P<0.008).

Supplementary Table S3. Comparison of soft drusen plus SDD in clusters 3 and 4.

	Soft drusen plus SDD in cluster 3 (N=8)	Soft drusen plus SDD in cluster 4 (N=13)	P*
Age (year), mean ± SD	73.8 ± 6.6	72.7 ± 7.5	0.64
Male/female (%)	0/8	6/7	0.02
Visual acuity (LogMAR)	0.05	0.08	0.90
Haller's vessels			
Diameter, mean (μm)	93.2 ± 12.5	82.5 ± 5.4	0.01
Diameter, SD (μm)	46.5 ± 23.0	34.5 ± 3.4	0.01
Diameter, maximum (μm)	342.0 ± 74.5	313.8 ± 55.8	0.31
Total vessel length (mm)	42.0 ± 13.9	58.3 ± 8.7	0.01
Total vessel area (mm^2)	7.1 ± 2.0	9.1 ± 1.1	0.01
Number of intersections	83.3 ± 28.8	173.5 ± 49.3	<0.001
Branch vessel length, mean (μm)	269.5 ± 17.6	208.5 ± 18.8	<0.001
Branch vessel length, SD (μm)	317.9 ± 35.4	210.2 ± 35.9	<0.001
Choriocapillaris flow voids			
Average size ($μm^2$)	546.0 ± 153.3	456.7 ± 85.1	0.15
Number	19816.5 ± 2678.7	21181.9 ± 1717.7	0.26
Total flow void area (mm^2)	10.5 ± 1.4	9.5 ± 1.0	0.11
Subfoveal CT (μm)	145 ± 50.2	139.9 ± 48	0.66

All values are presented as the mean ± standard deviation (SD).
SDD, subretinal drusenoid deposit; LogMAR, logarithm of the minimum angle of resolution; CT, choroidal thickness.
* P-value from Mann-Whitney test (sex ratio was compared by chi-square test).

Supplementary Table S4. Intergrader agreement on the parameters from en face Haller vessel images.

	ICC	P
Diameter, mean	0.92	<0.001
Diameter, SD	0.93	<0.001
Diameter, maximum	0.86	0.003
Total vessel length	0.75	0.03
Total vessel area	0.88	0.002
Number of intersections	0.78	0.02
Branch vessel length, mean	0.73	0.03
Branch vessel length, SD	0.71	0.04
Subfoveal CT	0.95	<0.001

ICC, intraclass correlation coefficient; CT, choroidal thickness.

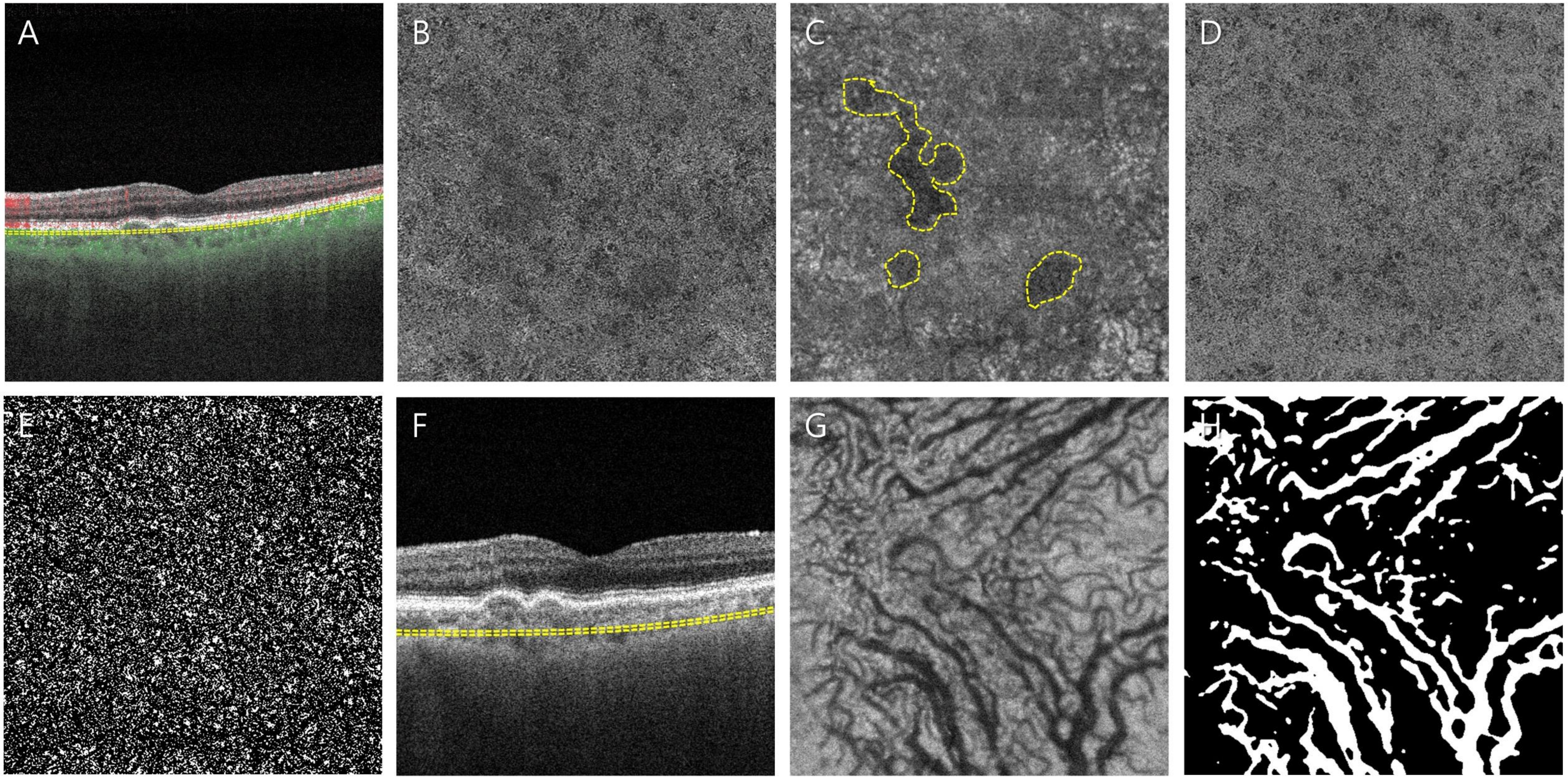

Supplementary Fig. S1. Signal compensation to enhance the attenuated signal of the choriocapillaris (CC) slab under drusen using structural en face optical coherence tomography (OCT). (A) Cross-sectional structural OCT image with soft drusen. Using a built-in segmentation algorithm for the CC layer (yellow dashed lines, thickness: 20 μm), an en face image of CC flow was generated. Red and green colors represent the flow signal over and under the retinal pigment epithelium, respectively. (B) OCT angiography (OCTA) image of the CC layer. (C) Structural en face OCT image of the same slab. Attenuated area by drusen is demarcated by yellow dotted line. (D) Compensated CC flow image obtained by structural OCT. The original OCTA image was multiplied by the inverted structural en face OCT image of the same slab. (E) Binarized flow voids from the compensated OCTA image of the CC layer for further quantitative analyses. (F) En face images of Haller's layer were obtained by locating the predefined slab of CC (yellow dashed lines, 20 μm thick) to the center of Haller's layer in B-scan mode scans. (G) En face structural OCT image of Haller's layer. (H) Binarized Haller's vessel image was prepared from the structural image for further quantitative analyses.

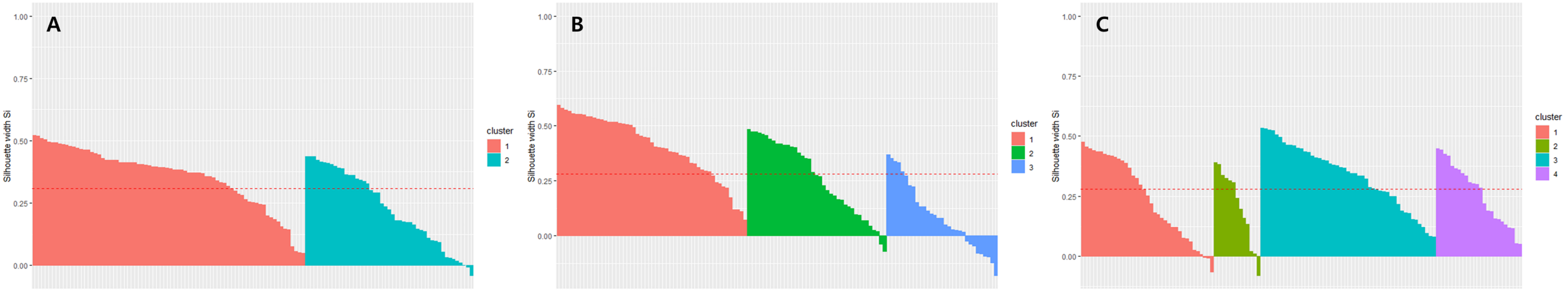

Supplementary Fig. S2. Silhouette plot depicting the silhouette width of each sample in the clusters. Graph showing the average silhouette width. The silhouette width displays how close each point in one cluster is to the points in the neighboring clusters. A high silhouette width indicates good clustering. The optimal number of clusters k is the number that maximizes the average silhouette of the clusters over a range of possible values for k and where the data points in each cluster have the highest possible positive silhouette value. Note the number of clusters (i.e., 2, 3, and 4 for A, B, and C, respectively) and their silhouette width in each cluster. The x-axis represents the eyes in each cluster, and the y-axis represents the silhouette width of each eye. Based on this plot, clusters 3 and 4 (B and C) have similarly higher average silhouette widths than cluster 2 (A). Cluster 4 (C) has fewer eyes with negative silhouette widths than cluster 3 (B). Therefore, the proposed number of clusters is 4.
